# 2-(4-Fluoro­phen­yl)-5-iodo-3-phenyl­sulfinyl-1-benzofuran

**DOI:** 10.1107/S1600536812013086

**Published:** 2012-03-27

**Authors:** Hong Dae Choi, Pil Ja Seo, Uk Lee

**Affiliations:** a Department of Chemistry, Dongeui University, San 24 Kaya-dong Busanjin-gu, Busan 614-714, Republic of Korea; b Department of Chemistry, Pukyong National University, 599-1 Daeyeon 3-dong, Nam-gu, Busan 608-737, Republic of Korea

## Abstract

In the title compound, C_20_H_12_FIO_2_S, the dihedral angles between the mean plane [r.m.s. deviation = 0.014 (1) Å] of the benzofuran fragment and the pendant 4-fluoro­phenyl and phenyl rings are 8.0 (1) and 86.06 (6)°, respectively. In the crystal, mol­ecules are linked by weak C—H⋯O hydrogen bonds. The crystal structure also exhibits weak π–π inter­actions between the furan and benzene rings of neighbouring mol­ecules [centroid–centroid distance = 3.547 (2) Å, inter­planar distance = 3.397 (2) Å and slippage = 1.021 (2) Å].

## Related literature

For background information and the crystal structures of related compounds, see: Choi *et al.* (2011 ▶); Seo *et al.* (2011 ▶).
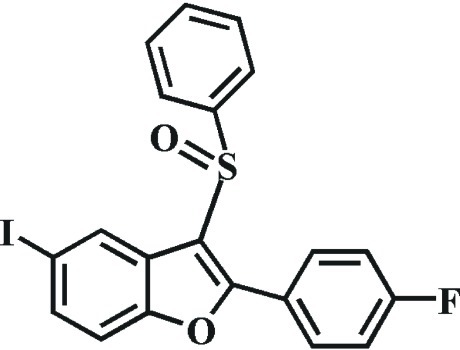


## Experimental

### 

#### Crystal data

C_20_H_12_FIO_2_S
*M* *_r_* = 462.26
Triclinic, 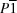

*a* = 8.1771 (2) Å
*b* = 9.8877 (2) Å
*c* = 11.8423 (3) Å
α = 103.108 (1)°
β = 90.872 (1)°
γ = 111.546 (1)°
*V* = 862.23 (4) Å^3^
*Z* = 2
Mo *K*α radiation
μ = 2.00 mm^−1^
*T* = 173 K
0.27 × 0.26 × 0.12 mm

#### Data collection

Bruker SMART APEXII CCD diffractometer
Absorption correction: multi-scan (*SADABS*; Bruker, 2009 ▶) *T*_min_ = 0.516, *T*_max_ = 0.746
15186 measured reflections
3974 independent reflections
3696 reflections with *I* > 2σ(*I*)
*R*_int_ = 0.026

#### Refinement

*R*[*F* ^2^ > 2σ(*F* ^2^)] = 0.024
*wR*(*F* ^2^) = 0.060
*S* = 1.07
3974 reflections
226 parameters
H-atom parameters constrained
Δρ_max_ = 0.50 e Å^−3^
Δρ_min_ = −0.75 e Å^−3^

### 

Data collection: *APEX2* (Bruker, 2009 ▶); cell refinement: *SAINT* (Bruker, 2009 ▶); data reduction: *SAINT*; program(s) used to solve structure: *SHELXS97* (Sheldrick, 2008 ▶); program(s) used to refine structure: *SHELXL97* (Sheldrick, 2008 ▶); molecular graphics: *ORTEP-3* (Farrugia, 1997 ▶) and *DIAMOND* (Brandenburg, 1998 ▶); software used to prepare material for publication: *SHELXL97*.

## Supplementary Material

Crystal structure: contains datablock(s) global, I. DOI: 10.1107/S1600536812013086/kj2197sup1.cif

Structure factors: contains datablock(s) I. DOI: 10.1107/S1600536812013086/kj2197Isup2.hkl

Supplementary material file. DOI: 10.1107/S1600536812013086/kj2197Isup3.cml

Additional supplementary materials:  crystallographic information; 3D view; checkCIF report

## Figures and Tables

**Table 1:** Hydrogen-bond geometry (Å, °)

*D*—H⋯*A*	*D*—H	H⋯*A*	*D*⋯*A*	*D*—H⋯*A*
C19—H19⋯O2^i^	0.95	2.38	3.297 (3)	163

Symmetry code: (i) 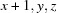.

**Fig. 1.**
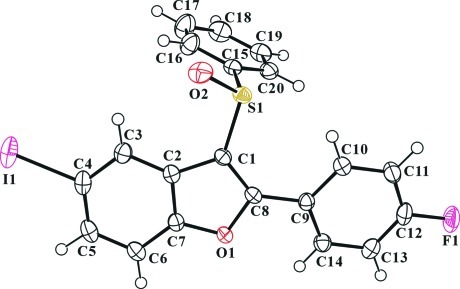
The molecular structure of the title compound with the atom numbering scheme. Displacement ellipsoids are drawn at the 50% probability level.

**Fig. 2.**
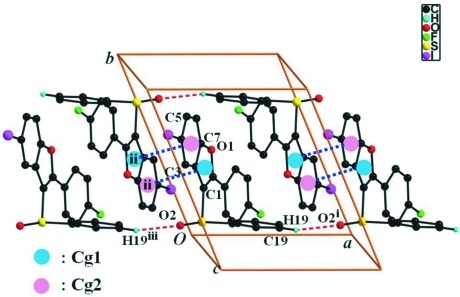
A view of the C—H···O and π–π interactions (dotted lines) in the crystal structure of the title compound. H atoms non-participating in hydrogen-bonding were omitted for clarity. [ Symmetry codes: (i) x + 1, y, z; (ii) - x, - y + 1, -z + 1; (iii) - x, - y + 1, - z + 1.]

## supplementary crystallographic information

### Comment

As a part of our ongoing study of 2-(4-fluorophenyl)-5-halo-3-phenylsulfinyl-1-benzofuran derivatives containing 5-chloro (Choi *et al.*, 2011) and 5-bromo (Seo *et al.*, 2011) substituents, we report herein the crystal structure of the title compound.

In the title molecule (Fig. 1), the benzofuran unit is essentially planar, with a mean deviation of 0.014 (1) Å from the least-squares plane defined by the nine constituent atoms. The dihedral angles between the mean plane of the benzofuran fragment and the pendant 4-fluorophenyl and phenyl rings are 8.0 (1)° and 86.06 (6)°, respectively. The crystal packing (Fig. 2) is stabilized by weak intermolecular C—H···O hydrogen bonds (Table 1). The crystal packing (Fig. 2) is further stabilized by weak π–π interactions between the furan and benzene rings of neighbouring molecules, with a Cg1···Cg2^ii^ distance of 3.547 (2) Å and an interplanar distance of 3.397 (2) Å resulting in a slippage of 1.021 (2) Å (Cg1 and Cg2are the centroids of the C1/C2/C7/O1/C8 furan ring and C2–C7 benzene ring, respectively).

### Experimental

77% 3-Chloroperoxybenzoic acid (179 mg, 0.8 mmol) was added in small portions to a stirred solution of 2-(4-fluorophenyl)-5-iodo-3-phenylsulfanyl-1-benzofuran (312 mg, 0.7 mmol) in dichloromethane (30 ml) at 273 K. After being stirred at room temperature for 4h, the mixture was washed with saturated sodium bicarbonate solution and the organic layer was separated, dried over magnesium sulfate, filtered and concentrated at reduced pressure. The residue was purified by column chromatography (hexane:ethyl acetate, 4:1 v/v) to afford the title compound as a colorless solid [yield 52%, m.p. 435–436 K; *R*_f_ = 0.49 (hexane:ethyl acetate, 4:1 v/v)]. Single crystals suitable for X-ray diffraction were prepared by slow evaporation of a solution of the title compound in ethyl acetate at room temperature.

### Refinement

All H atoms were positioned geometrically and refined using a riding model, with C—H = 0.95 Å (C-aromatic). Uiso(H) =1.2*U*_eq_(C-aromatic).

### Crystal data

**Table:**

C_20_H_12_FIO_2_S	*Z* = 2
*M**_r_* = 462.26	*F*(000) = 452
Triclinic, *P*1	*D*_x_ = 1.780 Mg m^−^^3^
Hall symbol: -P 1	Mo *K*α radiation, λ = 0.71073 Å
*a* = 8.1771 (2) Å	Cell parameters from 9967 reflections
*b* = 9.8877 (2) Å	θ = 2.5–27.5°
*c* = 11.8423 (3) Å	µ = 2.00 mm^−^^1^
α = 103.108 (1)°	*T* = 173 K
β = 90.872 (1)°	Block, colourless
γ = 111.546 (1)°	0.27 × 0.26 × 0.12 mm
*V* = 862.23 (4) Å^3^	

### Data collection

**Table:**

Bruker SMART APEXII CCD diffractometer	3974 independent reflections
Radiation source: rotating anode	3696 reflections with *I* > 2σ(*I*)
Graphite multilayer monochromator	*R*_int_ = 0.026
Detector resolution: 10.0 pixels mm^-1^	θ_max_ = 27.5°, θ_min_ = 1.8°
φ and ω scans	*h* = −10→10
Absorption correction: multi-scan (*SADABS*; Bruker, 2009)	*k* = −12→12
*T*_min_ = 0.516, *T*_max_ = 0.746	*l* = −15→15
15186 measured reflections	

### Refinement

**Table:**

Refinement on *F*^2^	Primary atom site location: structure-invariant direct methods
Least-squares matrix: full	Secondary atom site location: difference Fourier map
*R*[*F*^2^ > 2σ(*F*^2^)] = 0.024	Hydrogen site location: difference Fourier map
*wR*(*F*^2^) = 0.060	H-atom parameters constrained
*S* = 1.07	*w* = 1/[σ^2^(*F*_o_^2^) + (0.0266*P*)^2^ + 0.5718*P*] where *P* = (*F*_o_^2^ + 2*F*_c_^2^)/3
3974 reflections	(Δ/σ)_max_ = 0.001
226 parameters	Δρ_max_ = 0.50 e Å^−^^3^
0 restraints	Δρ_min_ = −0.75 e Å^−^^3^

### Special details

**Table:**

Geometry. All esds (except the esd in the dihedral angle between two l.s. planes) are estimated using the full covariance matrix. The cell esds are taken into account individually in the estimation of esds in distances, angles and torsion angles; correlations between esds in cell parameters are only used when they are defined by crystal symmetry. An approximate (isotropic) treatment of cell esds is used for estimating esds involving l.s. planes.
Refinement. Refinement of F^2^ against ALL reflections. The weighted R-factor wR and goodness of fit S are based on F^2^, conventional R-factors R are based on F, with F set to zero for negative F^2^. The threshold expression of F^2^ > 2sigma(F^2^) is used only for calculating R-factors(gt) etc. and is not relevant to the choice of reflections for refinement. R-factors based on F^2^ are statistically about twice as large as those based on F, and R- factors based on ALL data will be even larger.

### Fractional atomic coordinates and isotropic or equivalent isotropic displacement parameters (Å^2^)

**Table:**

	*x*	*y*	*z*	*U*_iso_*/*U*_eq_	
I1	0.02004 (2)	0.736536 (19)	0.939462 (12)	0.04406 (7)	
S1	0.04558 (6)	0.18031 (5)	0.55834 (4)	0.02240 (10)	
F1	0.4764 (2)	0.12714 (16)	0.06431 (11)	0.0421 (3)	
O1	0.29632 (18)	0.56238 (13)	0.46924 (11)	0.0224 (3)	
O2	−0.10716 (18)	0.17443 (17)	0.62692 (14)	0.0317 (3)	
C1	0.1437 (2)	0.36524 (19)	0.54025 (15)	0.0203 (3)	
C2	0.1408 (2)	0.4983 (2)	0.61933 (15)	0.0212 (3)	
C3	0.0716 (3)	0.5302 (2)	0.72497 (16)	0.0252 (4)	
H3	0.0036	0.4528	0.7598	0.030*	
C4	0.1067 (3)	0.6796 (2)	0.77636 (17)	0.0283 (4)	
C5	0.2028 (3)	0.7961 (2)	0.72598 (18)	0.0296 (4)	
H5	0.2227	0.8971	0.7644	0.035*	
C6	0.2688 (3)	0.7648 (2)	0.62058 (17)	0.0267 (4)	
H6	0.3329	0.8419	0.5843	0.032*	
C7	0.2368 (2)	0.6155 (2)	0.57083 (15)	0.0217 (3)	
C8	0.2380 (2)	0.40854 (19)	0.45144 (15)	0.0203 (3)	
C9	0.2959 (2)	0.3331 (2)	0.34844 (15)	0.0212 (3)	
C10	0.2368 (3)	0.1764 (2)	0.31272 (17)	0.0278 (4)	
H10	0.1543	0.1169	0.3549	0.033*	
C11	0.2971 (3)	0.1071 (2)	0.21679 (18)	0.0317 (4)	
H11	0.2572	0.0007	0.1929	0.038*	
C12	0.4154 (3)	0.1950 (2)	0.15691 (16)	0.0289 (4)	
C13	0.4753 (3)	0.3489 (2)	0.18758 (17)	0.0283 (4)	
H13	0.5565	0.4068	0.1439	0.034*	
C14	0.4147 (3)	0.4179 (2)	0.28375 (16)	0.0248 (4)	
H14	0.4545	0.5244	0.3059	0.030*	
C15	0.2209 (2)	0.1910 (2)	0.65768 (16)	0.0226 (4)	
C16	0.2111 (3)	0.2277 (3)	0.77601 (18)	0.0346 (5)	
H16	0.1115	0.2458	0.8053	0.042*	
C17	0.3483 (4)	0.2380 (3)	0.8519 (2)	0.0433 (6)	
H17	0.3428	0.2630	0.9337	0.052*	
C18	0.4935 (3)	0.2120 (3)	0.8089 (2)	0.0386 (5)	
H18	0.5883	0.2211	0.8613	0.046*	
C19	0.5009 (3)	0.1729 (2)	0.6898 (2)	0.0330 (4)	
H19	0.6001	0.1541	0.6605	0.040*	
C20	0.3635 (3)	0.1612 (2)	0.61308 (18)	0.0275 (4)	
H20	0.3670	0.1331	0.5312	0.033*	

### Atomic displacement parameters (Å^2^)

**Table:**

	*U*^11^	*U*^22^	*U*^33^	*U*^12^	*U*^13^	*U*^23^
I1	0.04049 (10)	0.06314 (12)	0.02678 (8)	0.02727 (8)	0.00553 (6)	−0.00508 (7)
S1	0.0202 (2)	0.0189 (2)	0.0272 (2)	0.00549 (17)	0.00357 (17)	0.00708 (16)
F1	0.0526 (9)	0.0506 (8)	0.0284 (6)	0.0319 (7)	0.0107 (6)	−0.0008 (6)
O1	0.0269 (7)	0.0166 (6)	0.0225 (6)	0.0077 (5)	0.0037 (5)	0.0036 (5)
O2	0.0197 (7)	0.0361 (8)	0.0429 (8)	0.0101 (6)	0.0104 (6)	0.0171 (6)
C1	0.0192 (8)	0.0192 (8)	0.0230 (8)	0.0079 (7)	0.0014 (7)	0.0050 (6)
C2	0.0194 (9)	0.0219 (8)	0.0220 (8)	0.0090 (7)	−0.0003 (7)	0.0030 (7)
C3	0.0221 (9)	0.0305 (10)	0.0235 (9)	0.0116 (8)	0.0023 (7)	0.0046 (7)
C4	0.0250 (10)	0.0359 (10)	0.0230 (9)	0.0159 (9)	−0.0008 (7)	−0.0020 (7)
C5	0.0302 (11)	0.0250 (9)	0.0311 (10)	0.0141 (8)	−0.0030 (8)	−0.0033 (7)
C6	0.0281 (10)	0.0208 (9)	0.0300 (9)	0.0099 (8)	0.0000 (8)	0.0032 (7)
C7	0.0218 (9)	0.0223 (8)	0.0213 (8)	0.0101 (7)	0.0009 (7)	0.0029 (7)
C8	0.0201 (9)	0.0167 (8)	0.0232 (8)	0.0068 (7)	−0.0004 (7)	0.0038 (6)
C9	0.0216 (9)	0.0226 (8)	0.0194 (8)	0.0097 (7)	0.0006 (7)	0.0035 (7)
C10	0.0340 (11)	0.0228 (9)	0.0260 (9)	0.0106 (8)	0.0054 (8)	0.0049 (7)
C11	0.0414 (12)	0.0258 (9)	0.0281 (10)	0.0166 (9)	0.0011 (9)	0.0008 (8)
C12	0.0311 (11)	0.0391 (11)	0.0200 (8)	0.0217 (9)	0.0011 (8)	−0.0001 (8)
C13	0.0260 (10)	0.0367 (10)	0.0231 (9)	0.0131 (9)	0.0042 (7)	0.0069 (8)
C14	0.0245 (9)	0.0236 (9)	0.0247 (9)	0.0082 (8)	0.0019 (7)	0.0044 (7)
C15	0.0209 (9)	0.0199 (8)	0.0281 (9)	0.0074 (7)	0.0039 (7)	0.0089 (7)
C16	0.0363 (12)	0.0484 (13)	0.0304 (10)	0.0265 (11)	0.0095 (9)	0.0134 (9)
C17	0.0509 (15)	0.0615 (15)	0.0276 (10)	0.0328 (13)	0.0023 (10)	0.0115 (10)
C18	0.0343 (12)	0.0424 (12)	0.0439 (13)	0.0185 (10)	−0.0033 (10)	0.0139 (10)
C19	0.0247 (10)	0.0329 (11)	0.0477 (12)	0.0148 (9)	0.0083 (9)	0.0157 (9)
C20	0.0280 (10)	0.0255 (9)	0.0328 (10)	0.0125 (8)	0.0089 (8)	0.0103 (8)

### Geometric parameters (Å, º)

**Table:**

I1—C4	2.096 (2)	C9—C10	1.399 (3)
S1—O2	1.4905 (15)	C10—C11	1.384 (3)
S1—C1	1.7716 (18)	C10—H10	0.9500
S1—C15	1.7978 (19)	C11—C12	1.371 (3)
F1—C12	1.353 (2)	C11—H11	0.9500
O1—C7	1.371 (2)	C12—C13	1.372 (3)
O1—C8	1.380 (2)	C13—C14	1.386 (3)
C1—C8	1.368 (3)	C13—H13	0.9500
C1—C2	1.440 (2)	C14—H14	0.9500
C2—C7	1.392 (3)	C15—C16	1.377 (3)
C2—C3	1.400 (3)	C15—C20	1.388 (3)
C3—C4	1.382 (3)	C16—C17	1.386 (3)
C3—H3	0.9500	C16—H16	0.9500
C4—C5	1.401 (3)	C17—C18	1.385 (4)
C5—C6	1.383 (3)	C17—H17	0.9500
C5—H5	0.9500	C18—C19	1.384 (3)
C6—C7	1.383 (3)	C18—H18	0.9500
C6—H6	0.9500	C19—C20	1.388 (3)
C8—C9	1.460 (2)	C19—H19	0.9500
C9—C14	1.397 (3)	C20—H20	0.9500
			
O2—S1—C1	107.18 (8)	C11—C10—H10	119.6
O2—S1—C15	106.52 (9)	C9—C10—H10	119.6
C1—S1—C15	97.14 (8)	C12—C11—C10	118.61 (19)
C7—O1—C8	106.99 (14)	C12—C11—H11	120.7
C8—C1—C2	107.66 (15)	C10—C11—H11	120.7
C8—C1—S1	126.91 (14)	F1—C12—C11	118.54 (19)
C2—C1—S1	125.42 (14)	F1—C12—C13	118.78 (19)
C7—C2—C3	119.34 (17)	C11—C12—C13	122.67 (18)
C7—C2—C1	104.82 (15)	C12—C13—C14	118.54 (19)
C3—C2—C1	135.82 (17)	C12—C13—H13	120.7
C4—C3—C2	116.96 (18)	C14—C13—H13	120.7
C4—C3—H3	121.5	C13—C14—C9	120.84 (18)
C2—C3—H3	121.5	C13—C14—H14	119.6
C3—C4—C5	122.95 (18)	C9—C14—H14	119.6
C3—C4—I1	118.98 (15)	C16—C15—C20	121.34 (18)
C5—C4—I1	118.05 (14)	C16—C15—S1	119.61 (15)
C6—C5—C4	120.26 (18)	C20—C15—S1	119.05 (15)
C6—C5—H5	119.9	C15—C16—C17	119.1 (2)
C4—C5—H5	119.9	C15—C16—H16	120.4
C7—C6—C5	116.56 (18)	C17—C16—H16	120.4
C7—C6—H6	121.7	C18—C17—C16	120.3 (2)
C5—C6—H6	121.7	C18—C17—H17	119.9
O1—C7—C6	125.34 (17)	C16—C17—H17	119.9
O1—C7—C2	110.74 (15)	C19—C18—C17	120.2 (2)
C6—C7—C2	123.92 (17)	C19—C18—H18	119.9
C1—C8—O1	109.79 (15)	C17—C18—H18	119.9
C1—C8—C9	135.62 (16)	C18—C19—C20	120.0 (2)
O1—C8—C9	114.52 (15)	C18—C19—H19	120.0
C14—C9—C10	118.46 (17)	C20—C19—H19	120.0
C14—C9—C8	119.83 (16)	C19—C20—C15	119.06 (19)
C10—C9—C8	121.71 (17)	C19—C20—H20	120.5
C11—C10—C9	120.86 (19)	C15—C20—H20	120.5
			
O2—S1—C1—C8	−153.19 (16)	C7—O1—C8—C9	−177.64 (15)
C15—S1—C1—C8	97.02 (18)	C1—C8—C9—C14	−170.7 (2)
O2—S1—C1—C2	28.24 (18)	O1—C8—C9—C14	5.9 (2)
C15—S1—C1—C2	−81.55 (17)	C1—C8—C9—C10	8.5 (3)
C8—C1—C2—C7	−0.1 (2)	O1—C8—C9—C10	−174.81 (17)
S1—C1—C2—C7	178.71 (13)	C14—C9—C10—C11	1.2 (3)
C8—C1—C2—C3	−178.5 (2)	C8—C9—C10—C11	−178.14 (18)
S1—C1—C2—C3	0.3 (3)	C9—C10—C11—C12	−0.3 (3)
C7—C2—C3—C4	−1.2 (3)	C10—C11—C12—F1	178.92 (18)
C1—C2—C3—C4	177.1 (2)	C10—C11—C12—C13	−0.6 (3)
C2—C3—C4—C5	1.6 (3)	F1—C12—C13—C14	−178.90 (18)
C2—C3—C4—I1	−176.41 (13)	C11—C12—C13—C14	0.6 (3)
C3—C4—C5—C6	−0.5 (3)	C12—C13—C14—C9	0.3 (3)
I1—C4—C5—C6	177.47 (15)	C10—C9—C14—C13	−1.1 (3)
C4—C5—C6—C7	−0.9 (3)	C8—C9—C14—C13	178.18 (17)
C8—O1—C7—C6	179.10 (18)	O2—S1—C15—C16	−15.33 (19)
C8—O1—C7—C2	0.1 (2)	C1—S1—C15—C16	95.01 (17)
C5—C6—C7—O1	−177.58 (18)	O2—S1—C15—C20	164.42 (15)
C5—C6—C7—C2	1.3 (3)	C1—S1—C15—C20	−85.24 (16)
C3—C2—C7—O1	178.77 (16)	C20—C15—C16—C17	1.3 (3)
C1—C2—C7—O1	0.0 (2)	S1—C15—C16—C17	−178.96 (18)
C3—C2—C7—C6	−0.3 (3)	C15—C16—C17—C18	0.2 (4)
C1—C2—C7—C6	−179.03 (18)	C16—C17—C18—C19	−1.2 (4)
C2—C1—C8—O1	0.1 (2)	C17—C18—C19—C20	0.7 (3)
S1—C1—C8—O1	−178.64 (13)	C18—C19—C20—C15	0.8 (3)
C2—C1—C8—C9	176.9 (2)	C16—C15—C20—C19	−1.8 (3)
S1—C1—C8—C9	−1.9 (3)	S1—C15—C20—C19	178.46 (15)
C7—O1—C8—C1	−0.1 (2)		

### Hydrogen-bond geometry (Å, º)

**Table:**

*D*—H···*A*	*D*—H	H···*A*	*D*···*A*	*D*—H···*A*
C19—H19···O2^i^	0.95	2.38	3.297 (3)	163

Symmetry code: (i) *x*+1, *y*, *z*.
